# Non-traumatic splenic rupture - a rare first presentation of diffuse large B-cell lymphoma and a review of the literature

**DOI:** 10.1186/s12885-018-4702-1

**Published:** 2018-08-02

**Authors:** Kogulakrishnan Kaniappan, Christopher Thiam Seong Lim, Pek Woon Chin

**Affiliations:** 1 Department of Internal Medicine, Hospital Enche Besar Hajjah Khalsom, Kluang, Johor, Malaysia; 2 Department of Medicine, Faculty of Medicine and Health Sciences, Universiti Putra Malaysia, 43400 Serdang, Malaysia

**Keywords:** Non-traumatic splenic rupture, Lymphoma, Non-Hodgkin’s lymphoma

## Abstract

**Background:**

Cases of non-traumatic splenic rupture are rare and entails a potentially grave medical outcome. Hence, it is important to consider the differential diagnosis of a non-traumatic splenic rupture in patients with acute or insidious abdominal pain. The incidence of rupture in Diffuse B-cell non-Hodgkin Lymphoma is highly infrequent (Paulvannan and Pye, Int J Clin Pract 57:245–6, 2003; Gedik et el., World J Gastroenterol 14:6711–6716, 2008), despite reports of various non-traumatic splenic rupture in the literature (Orloff and Peksin, Int Abstr Surg 106:1-11, 1958; Paulvannan and Pye, Int J Clin Pract 57:245–6, 2003). In this article, we attempt to highlight the features of a rare cause of splenic rupture that might serve as a future reference point for the detection of similar cases during routine clinical practice.

**Case presentation:**

A 40-year-old man presented with 1 week history of left hypochondriac pain associated with abdominal distention. There was no history of preceding trauma or fever. Clinical examination revealed signs of tachycardia, pallor and splenomegaly. He had no evidence of peripheral stigmata of chronic liver disease. In addition, haematological investigation showed anemia with leucocytosis and raised levels of lactate dehydrogenase enzyme. However, peripheral blood film revealed no evidence of any blast or atypical cells. In view of these findings, imaging via ultrasound and computed tomography of the abdomen was performed. The results of these imaging tests showed splenic collections that was suggestive of splenic rupture and hematoma. Patient underwent emergency splenectomy and the histopathological report confirmed the diagnosis as DLBCL.

**Conclusions:**

The occurrence of true spontaneous splenic rupture is uncommon. In a recent systematic review of 613 cases of splenic rupture, only 84 cases were secondary to hematological malignancy. Acute leukemia and non-Hodgkin lymphoma were the most frequent causes of splenic rupture, followed by chronic and acute myelogeneous leukemias. At present, only a few cases of diffuse large B-cell lymphoma (DLBCL) have been reported. The morbidity and mortality rate is greatly increased when there is a delay in the diagnosis and intervention of splenic rupture cases. Hence, there should be an increased awareness amongst both physicians and surgeons that a non-traumatic splenic rupture could be the first clinical presentation of a DLBCL.

## Background

Non-traumatic splenic rupture is a rare clinical presentation with potentially grave medical outcome. Owing to its elusive nature, the recognition of a non-traumatic splenic rupture requires a high index of clinical suspicion [1, 2]. Few incidences of true spontaneous rupture of spleen have been reported in the literature despite its rarity [3, 4]. Conversely, non-traumatic splenic rupture is common and often related to (also known as pathological rupture) a diseased spleen. Common causes of non traumatic splenic rupture include myeloproliferative diseases, vasculitis and infections (such as malaria or infectious mononucleosis). However, diffuse large B-cell lymphoma (DLBCL) remains an obscure cause of splenic rupture that requires unique attention [4, 5].

## Case presentation

A 40 year old Malay male was seen at the emergency department with 1 week history of left hypochondriac pain with concurrent abdominal distention. He also complained of loss of appetite and feeling lethargic for 1 month duration. He had no fever, nausea, vomiting, changes in bowel habits or any history of bleeding diathesis. There was no history of trauma. Neither there were any significant past medical history nor family history of malignancy. He was an active smoker for 20 years but denied any alcohol consumption or substance abuse.

On clinical examination, he was afebrile, with an elevated heart rate of 110 beats per minute and a blood pressure measurement of 121/79 mmHg. Patient appeared pale. Abdominal examination revealed enlarged, non-tender liver and spleen. There was no ascites or peripheral lymphadenopathy. Cardiovascular and respiratory examinations were otherwise unremarkable.

**Fig. 1:**
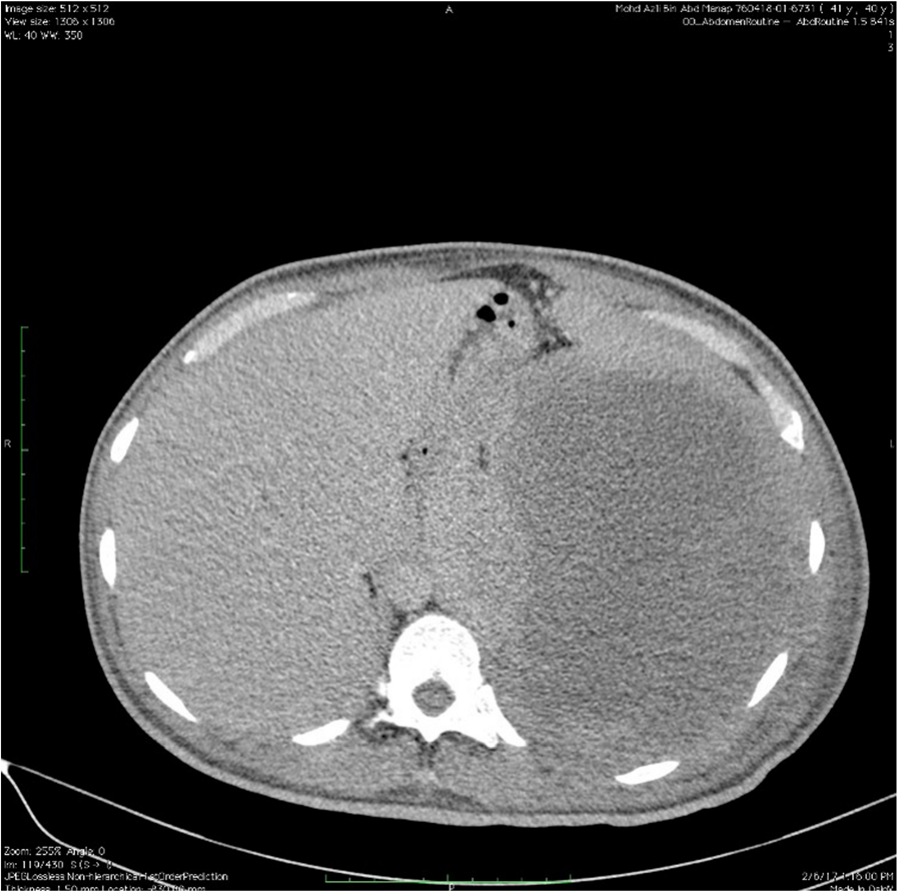
Contrast enhanced Computed Tomography of Thorax, Abdomen and Pelvis showing the abnormal spleen

**Fig. 2:**
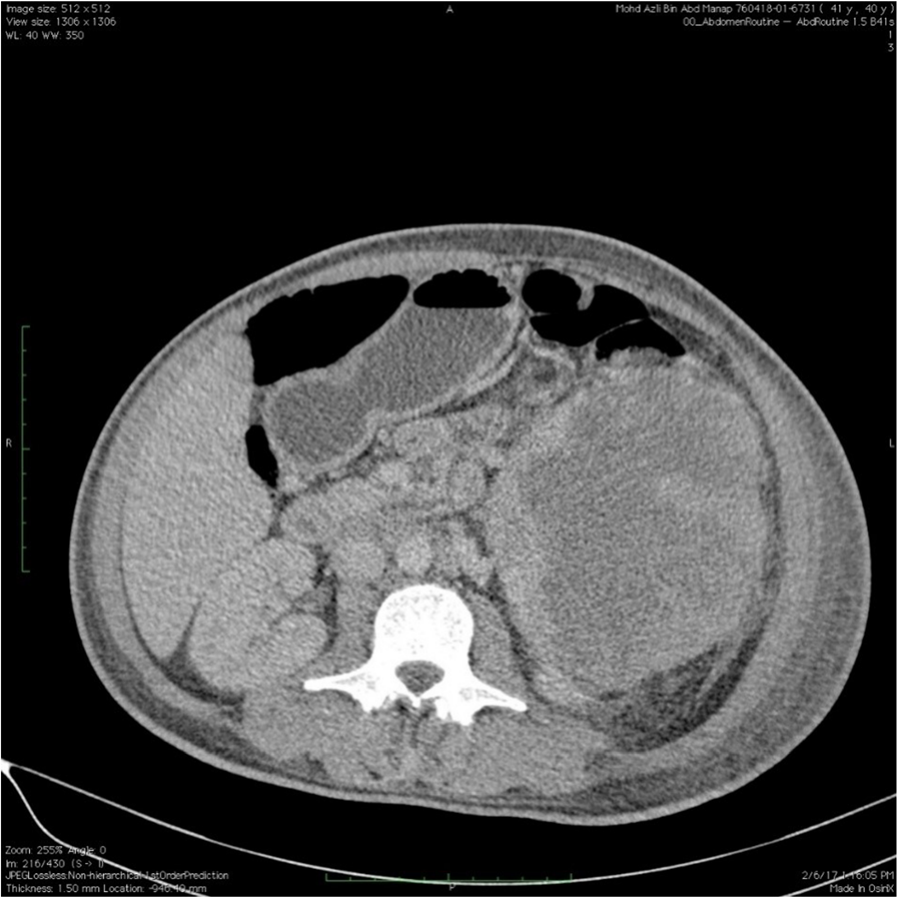
Contrast enhanced Computed Tomography of Thorax, Abdomen and Pelvis showing the abnormal spleen

**Fig. 3:**
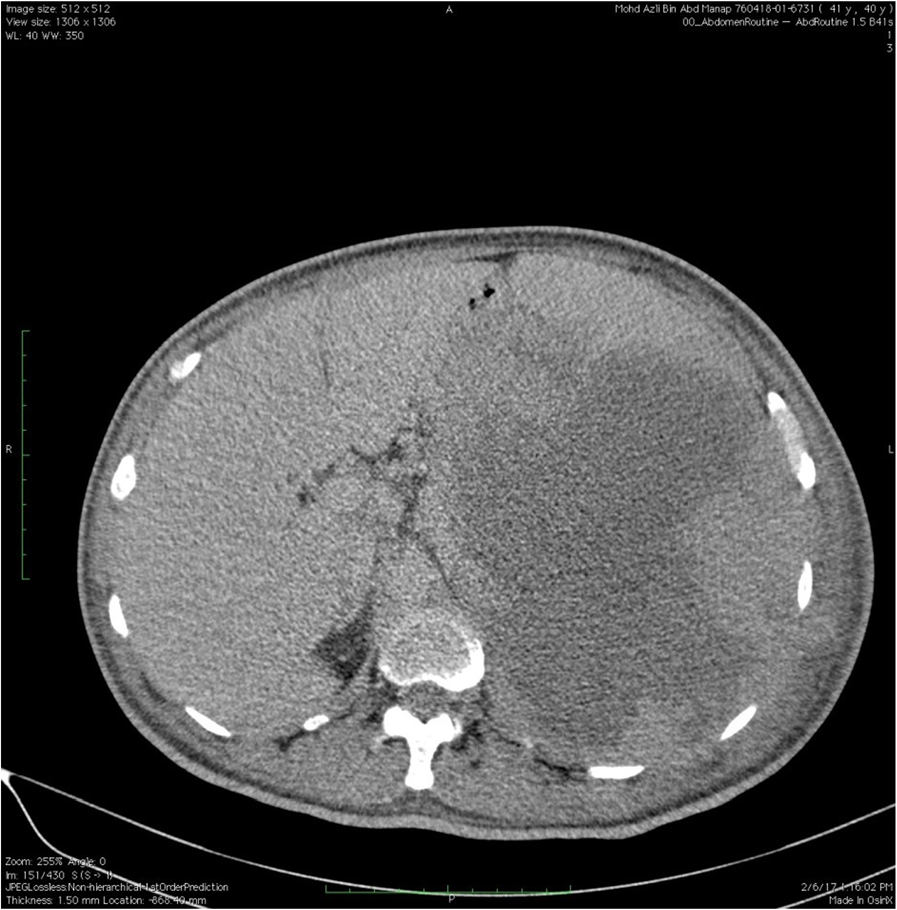
Contrast enhanced Computed Tomography of Thorax, Abdomen and Pelvis showing the abnormal spleen

Haematological investigation revealed a low haemoglobin level at 6.4 g/dl. The patient had a white cell count (WCC) of 33.3 × 10^^3^ /uL and a platelet count of 568 × 10^^3^/uL. Differential WCC showed a predominant neutrophil count of 79.9%, lymphocyte count 8.9%, monocytes 9.6%, eosinophils 0.8%, basophils 0.8%, absolute neutrophil count of 25.63 × 10^^3^ /uL and absolute lymphocyte count of 2.95 × 10^^3^ /uL. There was an increase in lactate dehydrogenase levels (LDH) from 534 to 666 u/L. Peripheral blood film revealed leucocytosis with neutrophilia with no evidence of blast cells or atypical lymphocytes. Patient was reluctant to undergo a bone marrow aspiration and trephine biopsy. Abdominal ultrasonography demonstrated a large splenic collection. A contrast enhanced computerized tomography of the abdomen further revealed a large heterogenous splenic collection measuring 18 cm × 15 cm × 16.9 cm which was suggestive of a splenic haematoma [Fig. 1, 2 and 3]. There were no intra abdominal or pelvic lymph nodes enlargement. Based on computed tomography findings, a preliminary diagnosis of spontaneous splenic rupture was made. A surgical consult was obtained and an explorative laparotomy was performed on the patient. Intra operative findings showed a ruptured spleen with extensive adhesions to the omentum. No intra peritoneal lymph nodes enlargement were found. Splenectomy was then performed and subsequently, the patient was transferred to intensive care unit for close observation.

**Fig. 4:**
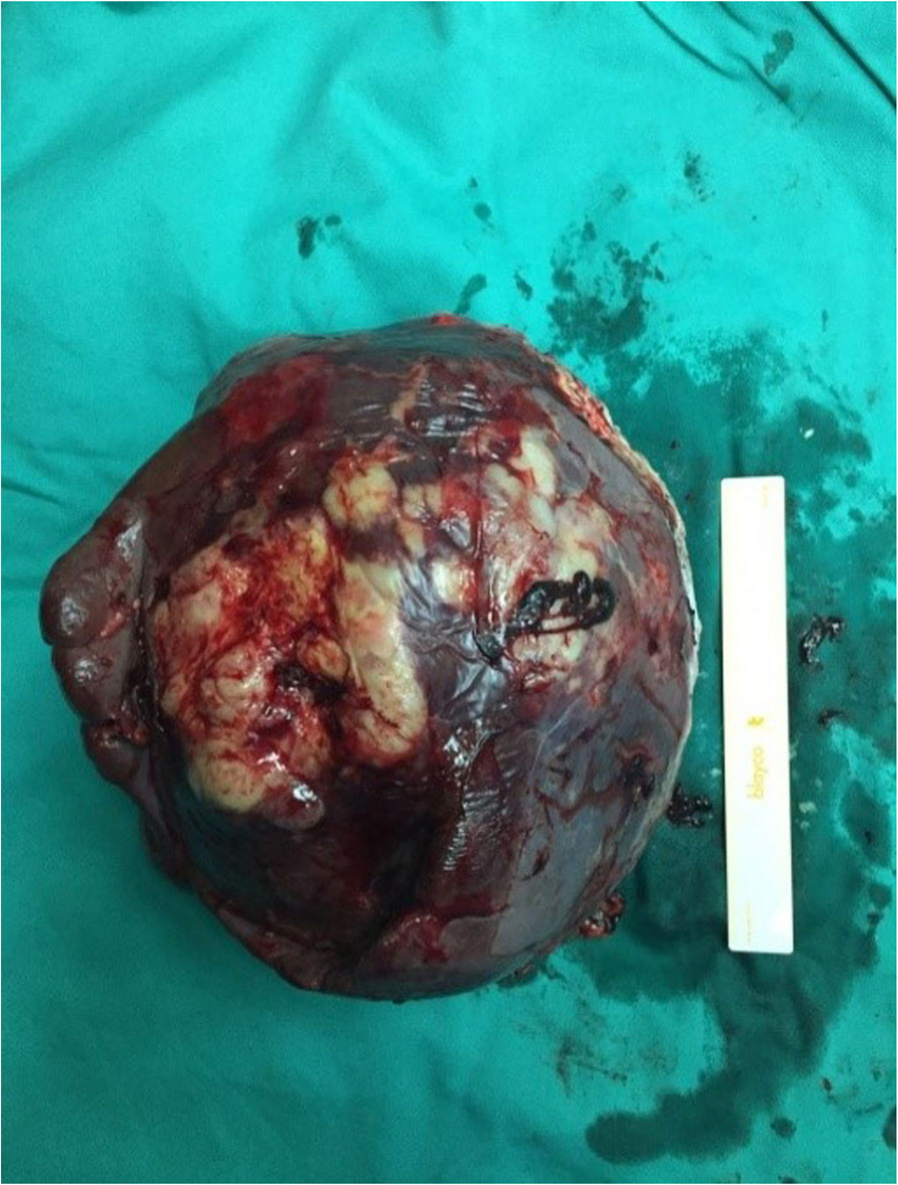
Intra operative image of the pathological spleen

From a histological perspective, the gross appearance of the obtained specimen revealed an enlarged spleen with irregular outer surfaces. A cut section of the spleen showed a firm, cream coloured layer occupying almost entire spleen with large area of necrosis with splenic infarcts. There as minimal amount of normal looking parenchyma tissues at the peripheral aspect of the specimen. Further histological examination revealed a diffuse infiltration of malignant lymphoid cells, which exhibited irregular nuclear membrane with vesicular nuclear chromatin and prominent nucleoli. The adjacent splenic parenchyma showed a congested and expanded red pulp with infiltration by atypical lymphoid cells [Fig. 4].

The histological report confirmed the presence of diffuse large B-cell non-Hodgkin’s lymphoma (NHL) via immunohistochemical testing. Immunohistochemical staining showed the cells to be positive to CD20 (diffuse), BCL-2, BCL-6 (> 30%), and MUM-1 (> 30%) and negative to CD3, CD10, Cyclin-D1, Tdt, CD30 and ALK. Ki67 proliferative index was > 80%. In accordance with the WHO classification of Lymphoid Neoplasm [6, 7], these findings were consistent with the diagnosis of a diffuse large B-cell non-Hodgkin’s lymphoma, non-germinal center B-cell (non-GCB) type. Further molecular studies to assess MYC / BCL-2 / BCL-6 translocation or rearrangement was not done as the fluorescence in situ hybridization (FISH) analysis was not available in our local laboratory settings. Thirteen days later, the patient was discharged with prophylactic meningococcal, pneumococcal and influenza vaccinations. He was referred to the haemato-oncologist outpatient clinic at a tertiary care centre for post - operative chemotherapy. Unfortunately, the patient did not turn up for subsequent follow ups, rendering it difficult to further document any information with regards to treatment response in this report.

## Discussion and conclusion

There is a variation in symptom manifestation in patients with splenic rupture. The presence of abdominal pain in splenic rupture has been frequently reported [8]. Abdominal pain, tenderness in the epigastrium and discomfort in the left upper quadrant may be seen in patients who has experienced minor injury [9]. In 20% of the cases, a sharp radiating pain to the left shoulder (Kehr’s sign) was observed [9]. In larger splenic injuries, signs of hypovolemic shock was a common presentation [10]. The clinical signs of shock include tachycardia, rapid breathing, paleness, reduced capillary filling time and hypotension [10]. In the absence of trauma, clinicians should exercise a high index of suspicion to rule out other rare causes of splenic rupture.

Bassler et al. documented 613 cases of splenic rupture ranging from cases without any antecedent cause to cases with the presence of obvious risk factors (Table 1). The aetiology of atraumatic splenic ruptures were listed in the decreasing order of prevalence as follows: infectious (mainly malaria and infectious mononucleosis), medical procedures related (mostly related to colonoscopy), haematological (commonly non-Hodgkin Lymphoma and Acute Lymphoblastic Leukemia), neoplastic disease, medication related (anti coagulation and thrombolytics), pregnancy-related and others. Majority of these cases had a haematological origin (13.7% of the reported cases) [11]. NHL was reported as the cause for splenic rupture in 6.3% of the reported cases of atraumatic splenic rupture [11]. Based on this systematic review, compounded with other evidence [4, 8, 12], we identified only a handful of splenic rupture that can be attributed to diffuse large B-cell lymphoma [13–33]. Other subtypes of non-Hodgkin lymphoma (splenic T cell lymphoma, blastic variant Mantle cell lymphoma, Mantle cell lymphoma, anaplastic large cell lymphoma, unspecified malignant lymphoma and hepatosplenic gamma delta T cell lymphoma) were reported with similar prevalence rates [13–33]. Cases of diffuse histiocytic lymphoma, follicular low grade lymphoma, malignant lymphomonocytic B-cell lymphoma and diffuse histiocytic lymphoma were reported infrequently [13–33].

**Table 1:** Overview of different causes of non-traumatic splenic rupture [11]

Categorization of causes	Number of cases reported (*n*)
Following a medical procedure	112
Infectious disease related	143
Haematological disease related	84
Rheumatological disease related	10
Pregnancy related	38
Non haematological neoplastic	48
Medication related	47
Internal trauma	17
Infiltrative disease	39
Related to splenic or adjacent physical abnormality	31
Miscellanous	44
TOTAL	613 CASES

In another review by P. Renzulli et al. [12] demonstrated a total of 845 patients who had experienced splenic rupture between the year 1980 to 2008. The six major aetiological groups were classified as follows: neoplastic (30.3%), infectious (27.3%), inflammatory, non-infectious (20%), drug or treatment related (9.2%), and normal spleen - idiopathic (7%) [12]. non-Hodgkin Lymphoma and acute Myeloid Leukemia were reported as a common finding among the neoplastic related atraumatic splenic ruptures [34]. The review also noted that the neoplastic subgroup was significantly associated with increased mortality rates [12, 34].

Non-traumatic splenic rupture secondary to haematological malignancies is still widely considered as an uncommon occurence [35]. Nonetheless, limited case reports advocate early recognition and intervention of this rare cause of splenic rupture [8, 36, 37]. In addition, several authors have attributed the low index of suspicion as a major reason for the delayed diagnosis of similar cases of spontaneous splenic rupture secondary to NHL [36, 38, 39]. To that effect, our case report presents rare descriptions where splenic rupture was detected as the first manifestation of a DLBCL [11, 13–33, 40–42].

Diffuse large B-cell lymphoma (DLBCL) is the commonest and also the most aggressive form of NHLs, accounting for at least 30% of the NHLs [43].The cancer of B lymphocytes can be fatal if left untreated [43]. Extranodal involvement is seen in only 30% of the cases [43]. Although it is thought to occur equally in all age groups, DLBCL appears to affect a predominantly middle age population [43]. Risk factors for developing DLBCL include evidence of family history of lymphoma, autoimmune disease, human immunodeficiency virus infection, hepatitis C virus seropositivity, high body mass and certain occupational exposures. The suggested mechanism of a non-traumatic splenic rupture in lymphoma include splenic enlargement, cellular infiltration, and eventual splenic infarction with associated capsular haemorrhage [44]. Although splenic enlargement itself poses the greatest risk for non-traumatic splenic rupture, there are also other factors that could possibly explain the reasons for a splenic rupture [44].

Initial presentation with B symptoms (fever, night sweats and significant weight loss) accounts for one third of the cases [45]. In most patients, the prevalent clinical findings are peripheral lymphadenopathy with an enlarged spleen [45]. Patients also can present with advanced extranodal disease on admission [45]. However, the combination of rare manifestation [splenic rupture, primary liver lymphoma and the presence of the Asian variant of intravascular lymphoma (AIVL)] often delays the diagnosis of the underlying malignancy [45].

The gold standard for the diagnosis diffuse large B-cell lymphoma (DLBCL) is dependent upon surgical excision biopsy of the lesion [45, 46]. Additional test that help the diagnosis include immunophenotyping using immunohistochemistry or flow cytometry or a combination of both techniques [46].The staging of NHL is in accordance to WHO classification of Lymphoid Neoplasm [6, 7]. At present, full contrast enhanced staging computerized tomography (CT) of the neck, chest, abdomen and pelvis is the modality of choice for staging such patients [46]. In addition, cases where central nervous system involvement (CNS) is suspected, lumbar puncture for cerebral fluid analysis is also required [46]. Recently, fluorodeoxyglucose positron emission tomography (FDG-PET) is strongly recommended as PET scan is more sensitive, especially for extra nodal disease and improve staging accuracy and subsequent response assessment[46–48].

In our case, patient’s immunohistochemical staining showed the cells to be positive to CD20 (diffuse), BCL-2, BCL-6 (> 30%), and MUM-1 (> 30%) and negative to CD3, CD10, Cyclin-D1, Tdt, CD30 and ALK. Ki67 proliferative index was > 80%. These findings were consistent with a diffuse large B-cell lymphoma, non-Germinal centre B-cell (non-GCB) type [45, 46]. Further molecular studies, using fluorescence in situ hybridization (FISH) analysis to study MYC and/or BCL-2 or BCL-6 translocation or rearrangement can help clinicians to decide most suitable chemotherapy regimes, as well as prognosticate patient outcome [49].

Chemotherapy forms the cornerstone in the therapy of B-cell lymphoma, both as curative and palliative options. Patients often present with disseminated disease upon diagnosis, making radiation therapy of limited efficacy at this stage of the disease [44–46].Chemotherapy regime such as R-CHOP (rituximab, cyclophosphamide, doxorubicin, vincristine, prednisolone) and R-ACVBP (rituximab, doxorubicin, cyclophosphamide, vindesine, bleomycin, prednisolone) are stratified according to age, international prognostic index and feasibility of dose -intensified approaches [44–46].The post therapy follow-up plays a vital role in evaluation of treatment response and possible relapses.

In summary, it is evident that a non-traumatic spontaneous splenic rupture is a rare first clinical manifestation of a DLBCL. In our case, the diagnosis was delayed and only confirmed via histopathological findings. Thus, in cases where the initial workup does not point towards a surgical cause, clinicians should raise the suspicion of an underlying malignancy. In such scenarios, urgent ultrasonography and contrast enhanced tomography are the ideal diagnostic modalities to avoid misdiagnosis and delay in intervention which can be fatal.

## Abbreviations

DLBCL: Diffuse large B-cell non-Hodgkin lymphoma
NHL: Non-Hodgkin’s lymphoma
